# Identification of IDH-mutant gliomas by a prognostic signature according to gene expression profiling

**DOI:** 10.18632/aging.101521

**Published:** 2018-08-15

**Authors:** Qiangwei Wang, Zhiliang Wang, Guanzhang Li, Chuanbao Zhang, Zhaoshi Bao, Zheng Wang, Gan You, Tao Jiang

**Affiliations:** 1 Beijing Neurosurgical Institute, Capital Medical University, Beijing, China; 2 Department of Neurosurgery, Beijing Tiantan Hospital, Capital Medical University, Beijing, China; 3 China National Clinical Research Center for Neurological Diseases, Beijing, China; 4 Center of Brain Tumor, Beijing Institute for Brain Disorders, Beijing, China; * Equal contribution

**Keywords:** IDH-mutant gliomas, mRNA, timeROC, prognostic signature

## Abstract

Background: Isocitrate dehydrogenase (IDH) mutations are the most common genetic aberrations in gliomagenesis. We aimed to build a high-efficiency prediction gene signature in patients with IDH-mutant glioma.

Methods: In total, 167 gliomas from Chinese Glioma Genome Atlas (CGGA) dataset were included for discovery. The Cancer Genome Atlas (TCGA) dataset was used for validation. R language was the main software environment for our statistical operation and graphics.

Results: We applied the Time-Dependent ROC Curve (timeROC) method to estimate the gene prediction accuracy of 3 years and 5 years in two datasets. Seven genes were selected for further analysis (AUC ≥ 0.7 in two datasets). A seven-gene enrichment score was established to predict the overall survival of 3 years and 5 years for IDH- mutant glioma patients. Moreover, the seven-gene signature was an independent prognostic indicator for patients with IDH-mutant glioma. Gene Ontology (GO) Analysis of associated genes revealed signature-related biological process of cell cycle and division.

Conclusion: We have identified a seven-gene signature that can provide a more accurate predictor of 3 years and 5 years for patients with IDH-mutant glioma. Moreover, the signature may potentially help neurosurgeons with the clinical personalized management of gliomas.

## Introduction

Gliomas, ranging from grade II -IV, are the most common malignant intracranial tumors. Modified therapy, combining radiotherapy with/without temozolomide in glioblastoma only increases the two-year survival rate to 27% [1,2]. According to the 2016 World Health Organization (WHO) Classification based on the mutation status of isocitrate dehydrogenase (IDH) and Chromosome 1p/19q status, the diffuse gliomas are mainly divided into five subtypes, Lower grade gliomas (LGG) with IDH-mutant and 1p/19q-codeleted subtype, LGG with IDH-mutant and 1p/19q-intact subtype, LGG with IDH-wildtype subtype, GBM with IDH-mutant subtype, and GBM with IDH-wildtype subtype [3].

Isocitrate dehydrogenase (IDH) is the key metabolic enzymes, converting isocitrate to α-ketoglutarate (αKG). IDH1/2 mutations specifically change the catalytic activity of enzyme, and directly catalyze α-ketoglutarate (α-KG) to R-2-hydroxyglutarate (R-2-HG). R-2-HG, which competitively inhibits multiple dioxygenase, initiates the occurrence of tumors [4]. IDH mutations have been discovered in plenty of tumors, covering adult acute myeloid leukemia (AML), intrahepatic cholangiocarcinoma, pheochromocytoma, etc [5–7]. Meanwhile, IDH mutations are present in nearly 80% of grade II~ III gliomas and secondary glioblastomas [8]. IDH mutations are stable markers to classify gliomas in progression and prognosis, and patients possessing IDH mutations have a significantly longer overall survival (OS) and progression free survival (PFS) in LGG and GBM [4]. However, as far as we know, patients with IDH-mutant glioma exhibited the heterogeneous clinical outcomes. Over the last two decades, genetic and molecular studies have identified several diagnostic and prognostic markers to stratify patients with IDH-mutant glioma. For example, IDH-mutant patients with 1p/19q co-deletion lived longer significantly [9] and ATRX mutation combined with IDH mutation was used to re-classify patients with astrocytic tumors [10–12]. In the present study, we developed a robust prognostic model to predict the overall survival of patients with IDH-mutant glioma. We obtained whole genome mRNA expression profiling data from Chinese Glioma Genome Atlas (CGGA) as training set and The Cancer Genome Atlas (TCGA) as validation set. By applying Time-Dependent receiver operating characteristic (timeROC) curve and GSVA method, we ascertained a seven-gene signature as an independent prognostic factor, which could accurately predict the 3 years and 5 years overall survival for patients with IDH-mutant glioma. This robust prognostic model provided a more comprehensive view for patients with IDH-mutant glioma and highlighted its potential role in the clinical management of gliomas.

## RESULTS

### Prognostic signature identified in IDH-mutant gliomas

In order to identify robust prognostic significance genes, we used time-dependent AUC to evaluate the gene’s prognostic accuracy of overall survival (3 years and 5 years) in CGGA and TCGA dataset, respectively. The genes with AUC in predicting overall survival (both 3 years and 5 years) more than 0.7 were considered as high prognostic factors. Finally, a total of 535 genes in CGGA dataset and 34 genes in TCGA dataset were selected (Table S1). Seven genes, shared by two datasets, were used to establish the signature in following analysis, including *WEE1, HOXD10, HOXD3, HOXD4, PRR11, HIST1H2BJ and IRX5* (Table 1).

**Table 1:** 7 genes with high prognostic value.

	CGGA (AUC≥0.8)		TCGA (AUC≥0.7)	
Time-dependent AUC	3 years	5 years	3 years	5 years
WEE1	0.869139	0.815010	0.868309	0.721559
HOXD10	0.817848	0.817430	0.756850	0.743001
HOXD3	0.887273	0.852331	0.713209	0.712660
HOXD4	0.881592	0.845084	0.764262	0.753305
PRR11	0.873778	0.920307	0.761877	0.725740
HIST1H2BJ	0.874227	0.802131	0.703374	0.703216
IRX5	0.819197	0.800984	0.753487	0.736913

**Figure 1:**
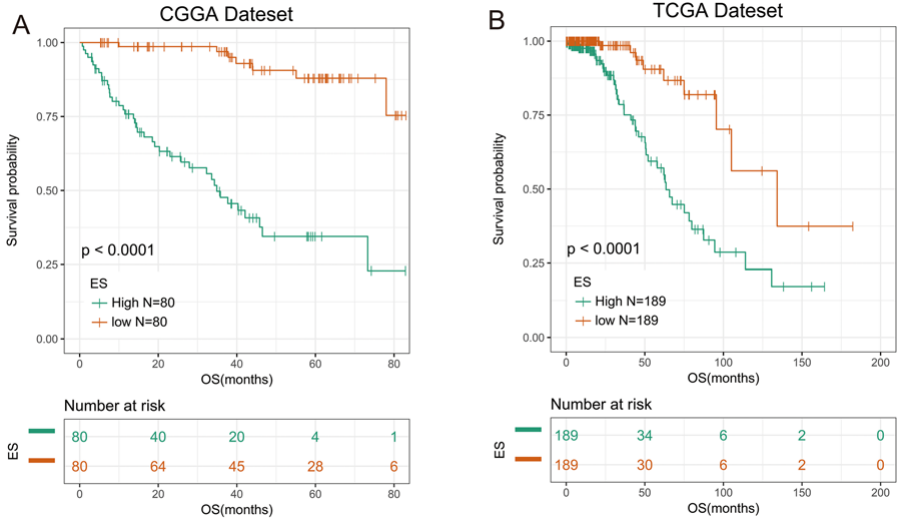
**7-gene signature had good prognostic value in training and validation data set.** (**A**) Kaplan–Meier survival analysis revealed high-ES group was worse in survival in CGGA data set. (**B**) Kaplan-Meier survival analysis proved high-ES was worse in survival in TCGA data set.

We then applied the seven genes to develop the enrichment score (ES) using the GSVA method. To assess the prognostic performance of ES, we calculated the ES for every patient in CGGA dataset and divided them into high-ES group and low-ES group based on the cutoff value (median enrichment score). We observed that the patients in high-ES group had a significant shorter overall survival than the low-ES group (*p* < 0.0001, Figure 1A).

### Validation of the prognostic value of the signature in TCGA dataset

**Figure 2:**
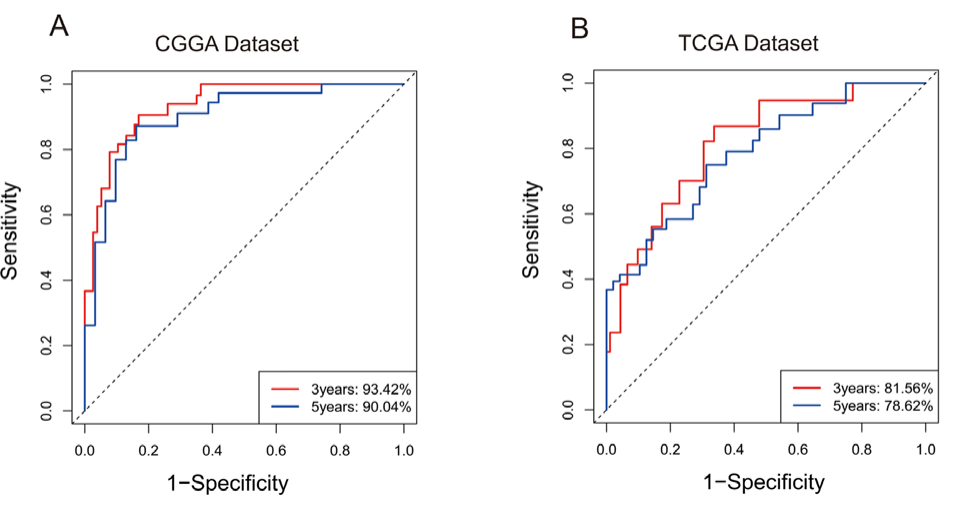
**7-gene signature predicted survival of 3 years and 5 years with high specificity and sensitivity.** (**A**) In CGGA, Area Under Curve (AUC) of 3 years and 5 years reached 93.42% and 90.04%. (**B**) In TCGA, Area Under Curve (AUC) of 3 years and 5 years reached 81.56% and 78.62%.

For the remaining 378 IDH-mutant glioma patients in TCGA dataset, we used the same method to calculate the enrichment score to validate the prognostic value of the seven-gene enrichment score. We divided patients into high-ES group and low-ES group according to the enrichment score (cutoff: median enrichment score). Ultimately, the result of the validation group was consistent with the training group (*p* < 0.0001, Figure 1B). Then we evaluated the prediction accuracy of ES of the overall survival. The ES showed higher time-dependent AUC in CGGA (3 years: 0.9342, 5 years: 0.9004) and TCGA (3 years: 0.8156, 5 years: 0.7862) dataset than seven genes individually (Figure 2A and B).

To determine whether the seven-gene enrichment score had prognostic value for IDH-wildtype patients, we selected 150 IDH-wildtype gliomas from CGGA and 225 IDH-wildtype gliomas from TCGA and calculated the ES for patients with the same method. A Kaplan-Meier analysis indicated that OS was reduced for high-risk patients with IDH-wildtype glioma (Figure S1A and B). The seven-gene enrichment score could reliably identify the high-risk patients both in patients with IDH-mutant and IDH-wildtype gliomas.

### Enrichment score was an independent prognostic factor for IDH-mutant patients

We conducted univariate and multivariate cox regression analysis to assess the prognostic value of our ES after adjusting previous widely accepted prognostic and predictive factors (gender, age, grade) in CGGA and TCGA datasets.

As shown in Table 2, in patients with IDH-mutant glioma, the grade and signature enrichment score were associated with survival in univariate analysis (p < 0.05). After adjusted with grade, enrichment score was still associated with survival significantly (p < 0.001). These results indicated that enrichment score was an independent prognostic factor for overall survival.

**Table 2:** Univariate and multivariate Cox regression analysis in two datasets.

	**Univariate**				**Multivariate**			
	**HR**	**95%CI**		**p**	**HR**	**95%CI**		**p**
		**Lower**	**Upper**			**lower**	**Upper**	
**CGGA dataset**								
Gender	0.939	0.525	1.682	0.833				
Age	1.517	0.752	3.060	0.244				
Grade	6.583	3.535	12.26	<0.001	2.746	1.445	5.220	0.002
Enrichment Score	10.14	4.496	22.88	<0.001	7.440	3.148	17.59	<0.001
**TCGA dataset**								
Gender	0.889	0.508	1.553	0.678				
Age	1.597	0.877	2.910	0.126				
Grade	3.468	1.055	11.4	0.041	2.163	0.653	7.171	0.207
Enrichment Score	4.332	2.104	8.918	<0.001	4.165	2.013	8.618	<0.001

In Cox regression analysis: gender was defined as 1, male, 0, female; age was defined as 1, >45, 0, ≤45;grade was defined as 1, IV, 0, II/III.

### Functional annotation of Enrichment Score

**Figure 3:**
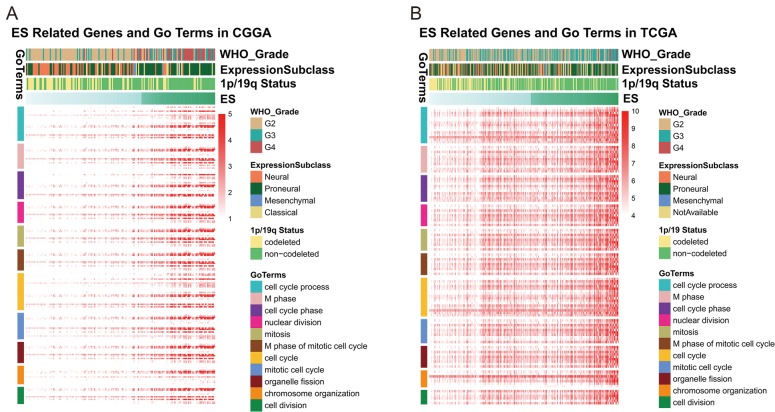
ES positively related genes were involved in biological process of cell cycle and division.

In order to further investigate the biological significance and explain the result above, we utilized Pearson correlation analysis to select genes that were strongly correlated with ES (Pearson R ≥ 0.5 in CGGA, Pearson R ≥ 0.4 in TCGA). A total of 637 and 344 genes in CGGA and TCGA dataset met the criteria, respectively (Table S2). We chose the overlapped genes (145 genes) in two datasets for Gene Ontology Analysis in DAVID and found positively related genes were basically enriched in biological functions of cell cycle and cell division (Figures 3A and B), partially explaining the malignancy and poor survival of patients in high-ES group. Meanwhile, we also noticed that 1p/19q co-deletion patients mainly focused on the low-risk group while the 1p/19q intact patients were contrary (CGGA: chi-square = 13.852, p-value = 0.0001977; TCGA: chi-square = 38.938, p-value = 3.505e-09). This result indicated that the ES was closely correlated with 1p/19q status.

## DISCUSSION

The 2016 World Health Organization Classification of Tumors of the Central Nervous System utilized molecular parameters and established the new concept of CNS tumor diagnosis in molecular era [3]. IDH-mutant glioma is an important entity ranging from WHO grade II to grade IV with different clinical manifestations, and objective molecular based classification is in urgent need.

The ROC curve accurately reflects the relationship between specificity and sensitivity of a continuous diagnostic marker. With time-dependent ROC method, Tada T et al detected that HBcrAg was an excellent predictive factor for hepatocellular carcinoma development throughout the follow-up period [13]. Piessevaux H et al revealed that Early Tumor Shrinkage (ETS) was a powerful clinical predictor for patients with metastatic colorectal cancer (mCRC) with cetuximab treatment [14].

Hence, we assumed that a group of genes could accurately predict the survival of patients with IDH-mutant glioma. With the method of time-dependent ROC, we performed gene expression analysis with overall survival in CGGA and TCGA datasets and successfully screened out seven genes with higher time- dependent AUC. Enrichment score (ES) based on seven genes was calculated for every patient and the high-ES group had a significantly poor survival. Utilizing enrichment score, we found time-dependent AUC of 3 years and 5 years was further improved. Univariate and multivariate Cox regression analysis proved that enrichment score was an independent prognostic factor after adjusting grade. However, the time-dependent AUC of 2 years and 3 years in patients with IDH-wildtype glioma (0.6880, 0.8218 in CGGA ; 0.6997, 0.7747 in TCGA; Figure S2A and B) were not as well as patients with IDH-mutant glioma. This result might ascribe to the different genetic backgrounds of gliomas. Through in-depth analysis of the ES-related biological functions, we found that ES was mainly related to cell mitosis and cell division.

The seven genes included in our signature were *WEE1, HOXD10, HOXD3, HOXD4, PRR11, HIST1H2BJ, IRX5.* To investigate the risk of seven genes, we constructed the heatmap based on gene expression profile. The heatmap showed that the expression of seven genes increased with ES except IRX5 in CGGA dataset and it’s identical in TCGA except WEE1 (Figures S3A and B). Hence, seven genes included in our signature were of high risk and patients with high expression would have a poor prognosis. As shown below, seven genes show some similarity in functions involved in cell mitosis, proliferation, metastasis and progression of tumors. WEE1 encodes a nuclear protein, which inhibits kinase activity of CDK1 and cell mitosis. Some studies found over-expression of WEE1 in gliomas and interfering with WEE1 expression has therapeutic potential in glioblastomas [15]. HOXD10 functions as a transcription factor and is related to some disease such as Wilm's tumor, congenital vertical talus and Charcot-Marie-Tooth disease. HOXD10 is associated with the progression of some tumors [16–18]. HOXD3 regulates cell adhesion processes and enhancing invasion and metastasis of tumor cells. Studies proved that HOXD3 expression was a significant unfavorable prognostic factor for tumor patients [19]. HOXD4 belongs to the homeobox family of genes and we can observe the change of HOXD4 in tumors like colon carcinoma [20] and breast cancer [21]. PRR11 is strictly regulated throughout the cell cycle. PRR11 is a candidate oncogene that has been implicated in the pathogenesis of lung cancer, gastric cancer and hilar cholangiocarcinoma [22–24]. HIST1H2BJ encodes a histone that is a member of H2B family. Four core histones (H2A, H2B, H3, and H4) form an octamer, around which DNA is wrapped in nucleosomes. IRX5 is also related to homeobox gene family and embryonic development. In prostate cancer, IRX5 play an vital role in regulating cell cycle and apoptosis and is expected to play a crucial part in tumor therapy [25]. Interestingly, three genes of the signature belong to the homeobox (HOX) family of genes. HOX is a class of genes that regulate the biological form in an organism, and once these genes mutate, it deforms part of the body. The mechanism of action is to regulate other genes related to cell division, direction of spindle, and the development of wirehair and appendage. Aberrant expression and carcinogenicity of HOX genes have been found in many solid tumors, such as colon, prostate, lung, bladder, ovarian, kidney, breast, etc. And proteins encoded by HOX genes play roles of either transcriptional activators or repressors, both promote oncogenesis [26]. There were also some studies on the interaction between HOX gene and glioma. Guo YB et al. found that inhibition expression of HOXA6 and B13 obviously reduced invasion of GBM U-118 and U-138 cells [27]. Duan R et al. transfected HOXA13 into glioma cells and it promoted proliferation and invasion of cells [28]. Tabuse M et al. silenced the expression of HOXD9 and detected the apoptosis of glioma cell [29]. With RT-PCR, Buccoliero AM et al. perceived the expression pattern of HOXD differed between low-grade gliomas and normal tissues [30]. Meanwhile, some HOX genes are closely related to patients’ survival [28,31].

In summary, we found a seven-gene enrichment score which could be a useful tool of risk stratification and predict the overall survival of 3 years and 5 years for patients with IDH-mutant glioma. These findings extend our understanding of the malignant progression in IDH-mutant gliomas and may potentially help neurosurgeons with the clinical personalized management of IDH-mutant glioma patients.

## MATERIALS AND METHODS

### Clinical characteristics of samples

In Chinese Glioma Genome Atlas (CGGA) dataset, we have collected mRNA sequencing data of 167 IDH-mutant glioma samples, ranging from WHO grade II to grade IV, generated with Illumina Hiseq platform. In the Cancer Genome Atlas (TCGA) dataset, mRNA-Seq data of 378 IDH-mutant glioma samples were downloaded online (https://cancergenome.nih.gov/). The clinical and molecular characteristics of those patients from two datasets were shown in Table 3. In CGGA, median age of patients was 38 (ranging from 10 to 62) and median age of TCGA was 39 (ranging from 14 to 75). The proportion of male was 101 (60%) in CGGA and 215 (57%) in TCGA. CGGA dataset included 94 (56%) patients with WHO Grade II glioma, 37 (22%) with Grade III glioma and 36 (22%) with GBM. TCGA included 195 (52%) patients with WHO Grade II glioma, 174 (46%) with Grade III glioma and 9 (2%) with Grade IV glioma. Chromosome 1p/19q co-deletion was detected in 64 (38%) patients in CGGA and 151 (40%) patients in TCGA. Meanwhile, MGMT promoter methylation was harbored in 88 (53%) patients in CGGA and 351 (93%) patients in TCGA. (MGMT promoter methylation status was not available in 53 (32%) patients in CGGA). Tumor tissue samples were obtained by surgical resection. All patients provided written informed consent, and the study was approved by the ethics committees of the participating hospitals. Survival data were collected by clinics during patient visits and/or phone interviews. Patients who underwent biopsy alone were not followed up at our center and were therefore excluded from the survival analysis.

**Table 3:** Baseline patient characteristics.

		CGGA(n=167,%)	TCGA(n=378,%)
Age	Median (range)	38(10-62)	39(14-75)
Gender	Male	101(60)	215(57)
	Female	66(40)	163(43)
KPS score	Preoperative KPS≥80	61(37)	193(51)
	Preoperative KPS<80	16(10)	27(7)
Pathological type	A	41(25)	116(31)
	O	34(20)	154(41)
	OA	56(34)	99(26)
	GBM	36(22)	9(2)
TCGA subtype	Neural	56(34)	77(20)
	Proneural	92(55)	199(53)
	Mesenchymal	4(2)	14(4)
	Classical	15(9)	0(0)
Grade	II	94(56)	195(52)
	III	37(22)	174(46)
	IV	36(22)	9(2)
1p19q status	codeletion	64(38)	151(40)
	non-codeletion	103(62)	226(60)
	NA	0(0)	1(0)
MGMT promoter methylation	Methylated	88(53)	351(93)
	Not methylated	26(16)	26(7)
	NA	53(32)	1(1)

### Detection of IDH1/2 mutations

In CGGA dataset, pyrosequencing technique is commonly used to detect IDH1/2 mutation [32]. And status of IDH1/2 mutation downloaded online from TCGA is mainly obtained through the method of whole exon sequencing (WES) or pyrosequencing.

### Gene Set Variation Analysis (GSVA)

The GSVA technique allows sensitive identification of differences in expression of predefined sets of genes between heterogeneous groups and can be used to explore underlying pathways. For functional annotation in this study, GSVA was used to calculate the enrichment score (ES) for every patient and build gene signature based on the expression of seven genes. ES values range from −1 to 1 [33].

### Statistical analysis

R, version 3.4.2 (http://www.r-project.org) is the main software environment for our statistical operation and graphics. Time-dependent ROC curve (timeROC) and Area under timeROC curve (AUC) were generated with R package “timeROC” [34]. Survival analysis and univariate and multivariate Cox regression analysis were performed with R package “survival”. Heatmap of ES related genes was drawn with R package “pheatmap”. P value ≤ 0.05 was considered to be statistically significant.

### Gene Ontology (GO) Analysis of Associated Genes

Significantly related genes that were shared by CGGA and TCGA dataset (145 genes) were chosen for Gene Ontology analysis in DAVID Bioinformatics Resources 6.8 (https://david.ncifcrf.gov/) for function annotation [35].

## SUPPLEMENTARY MATERIAL

Table S1

Table S2

Figure S1

Figure S2

Figure S3
