# Supplementary material for: Identification of IDH-mutant gliomas by a prognostic signature according to gene expression profiling
Source: Aging (Albany NY). 2018 Aug 15;10(8):1977–88. doi: 10.18632/aging.101521 (PMC6128431; doi:10.18632/aging.101521)
Supplement: Figure S1 [file aging-10-101521-s003.docx]

**
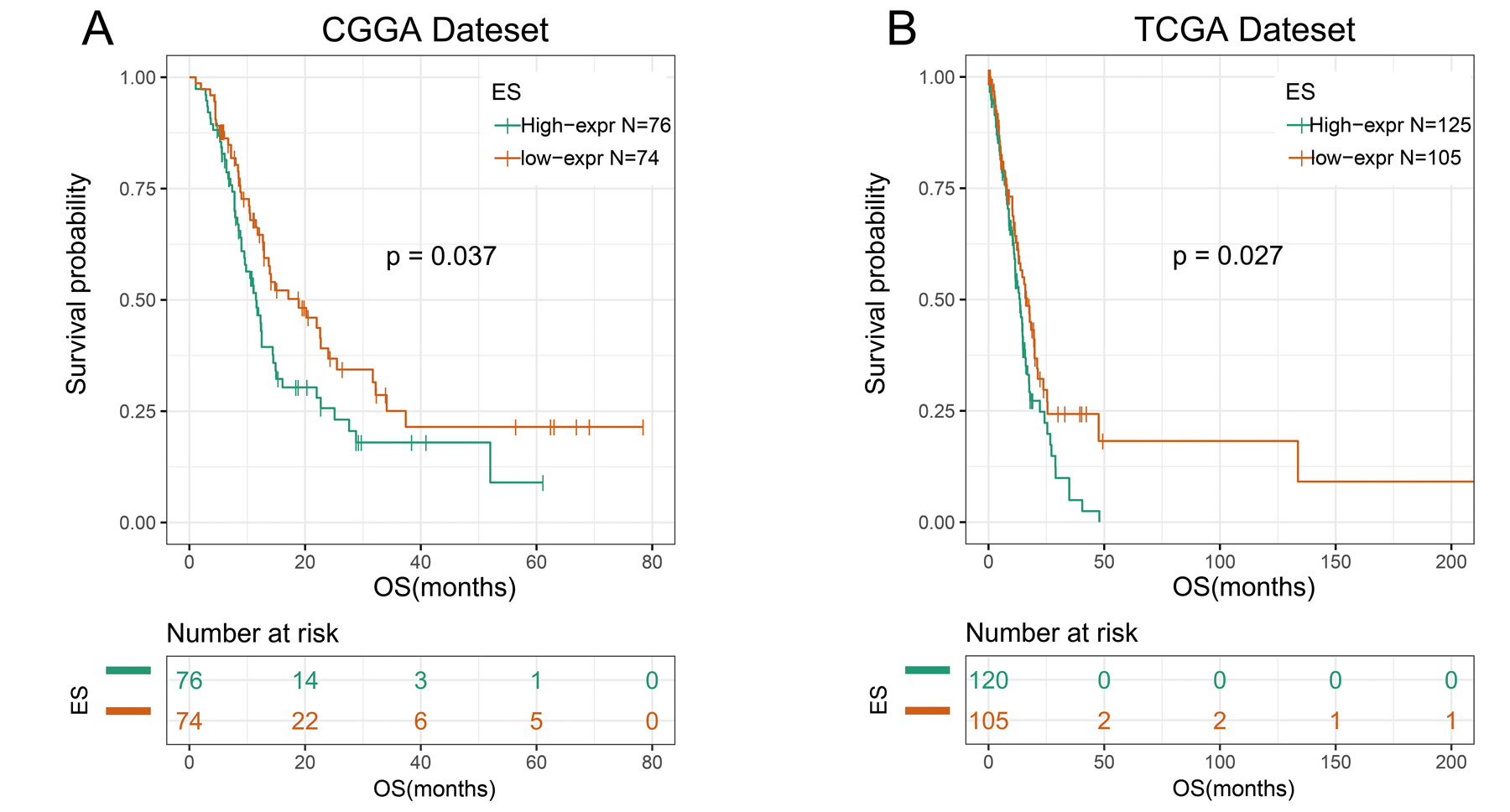
**

**Figure S1. 7-gene signature had good prognostic value in IDH-wildtype gliomas.** (**A**) Kaplan-Meier survival analysis revealed high-ES group was worse in survival in CGGA dataset. (**B**) Kaplan-Meier survival analysis proved high-ES was worse in survival in TCGA dataset.
