# Supplementary material for: Identification of IDH-mutant gliomas by a prognostic signature according to gene expression profiling
Source: Aging (Albany NY). 2018 Aug 15;10(8):1977–88. doi: 10.18632/aging.101521 (PMC6128431; doi:10.18632/aging.101521)
Supplement: Figure S2 [file aging-10-101521-s004.docx]

**
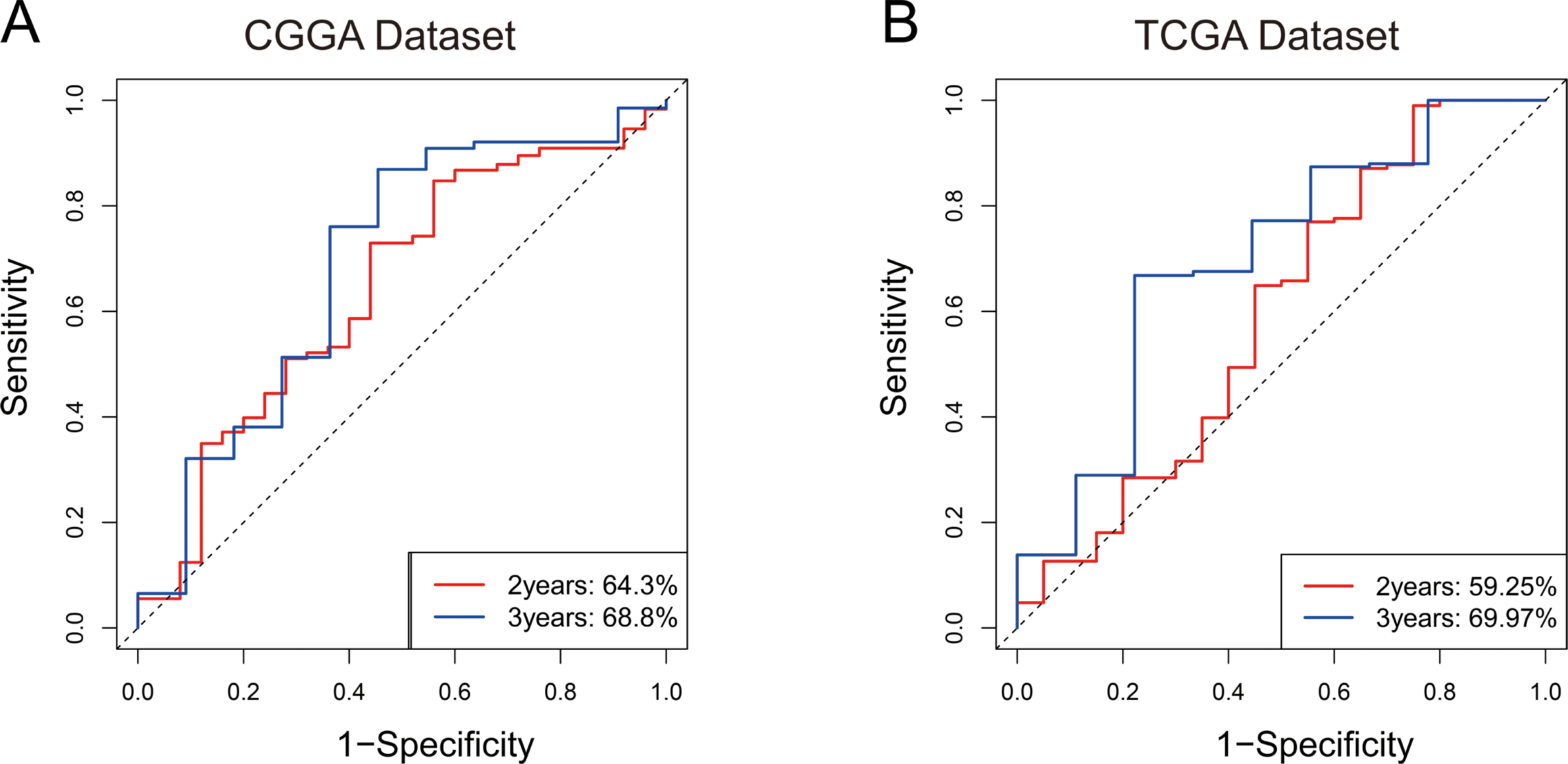
**

**Figure S2. 7-gene signature predicted survival of 2 years and 3 years in IDH-wildtype gliomas.** (**A**) In CGGA, Area Under Curve (AUC) of 2 years and 3 years reached 64.3% and 68.8%. (**B**) In TCGA, Area Under Curve (AUC) of 2 years and 3 years reached 59.25% and 69.97%.
