# Supplementary material for: Identification of IDH-mutant gliomas by a prognostic signature according to gene expression profiling
Source: Aging (Albany NY). 2018 Aug 15;10(8):1977–88. doi: 10.18632/aging.101521 (PMC6128431; doi:10.18632/aging.101521)
Supplement: Figure S3 [file aging-10-101521-s005.docx]

**
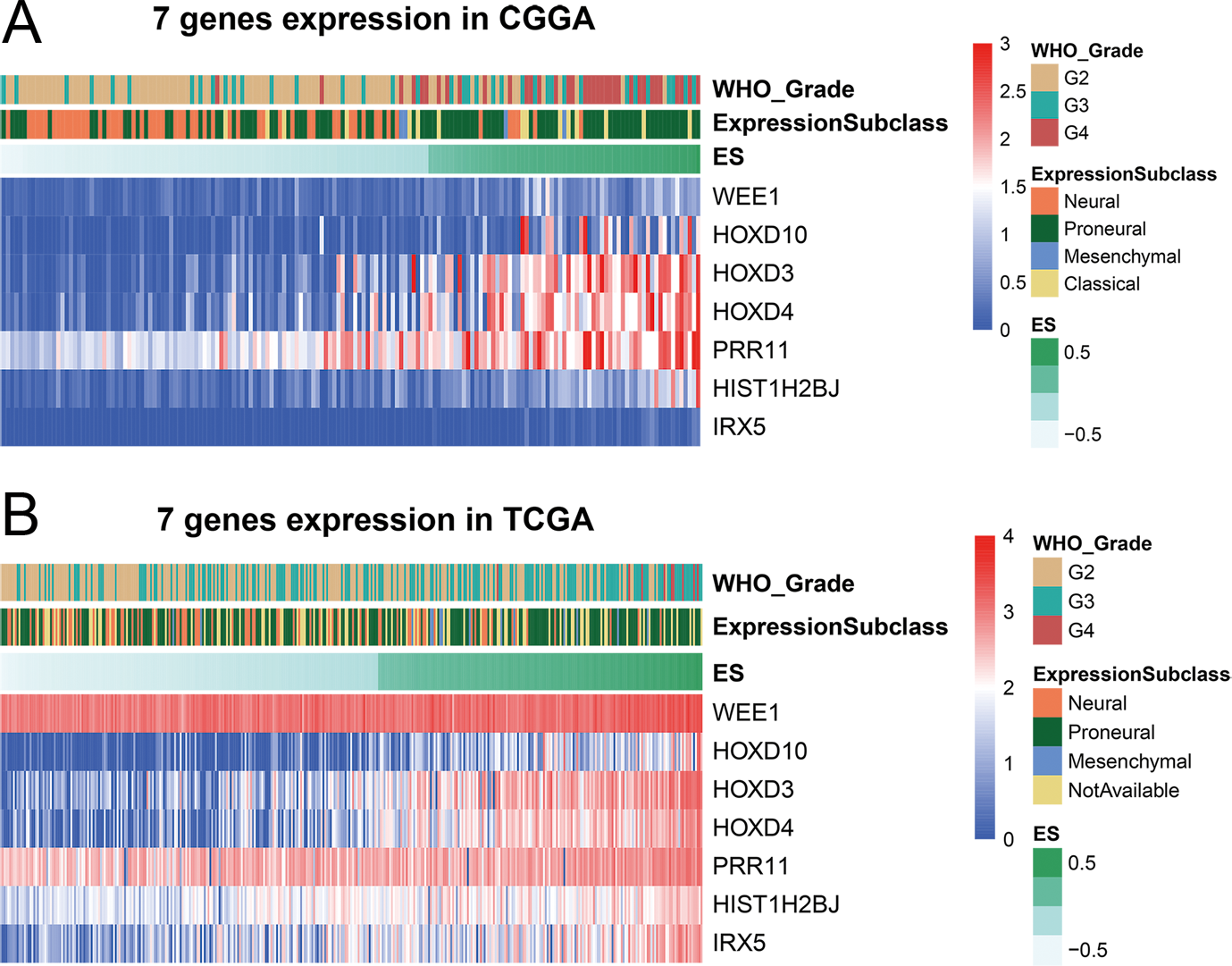
**

**Figure S3. Heatmap based on seven gene expression profile.** Seven genes increased with ES in CGGA and TCGA dataset.
